# SwinCVS: a unified approach to classifying critical view of safety structures in laparoscopic cholecystectomy

**DOI:** 10.1007/s11548-025-03354-9

**Published:** 2025-04-11

**Authors:** Franciszek M. Nowak, Evangelos B. Mazomenos, Brian Davidson, Matthew J. Clarkson

**Affiliations:** 1 UCL Hawkes Institute, Department of Medical Physics and Biomedical Engineering, UCL, London, UK; 2 Division of Surgery and Interventional Science, UCL, London, UK

**Keywords:** Critical view of safety, Laparoscopic cholecystectomy, Machine learning

## Abstract

**Purpose:**

Laparoscopic cholecystectomy is one of the most commonly performed surgeries in the UK. Despite its safety, the volume of operations leads to a notable number of complications, with surgical errors often mitigated by the critical view of safety (CVS) technique. However, reliably achieving CVS intraoperatively can be challenging. Current state-of-the-art models for automated CVS evaluation rely on complex, multistage training and semantic segmentation masks, restricting their adaptability and limiting further performance improvements.

**Methods:**

We propose SwinCVS, a spatiotemporal architecture designed for end-to-end training. SwinCVS combines the SwinV2 image encoder with an LSTM for robust CVS classification. We evaluated three different backbones—SwinV2, VMamba, and ResNet50—to assess their ability to encode surgical images. SwinCVS model was evaluated with the end-to-end variant, and with the pretrained variant with performance statistically compared with the current state-of-the-art, SV2LSTG on Endoscapes dataset.

**Results:**

SwinV2 demonstrated as the best encoder achieving +2.07% and +17.72% mAP over VMamba and ResNet50, respectively. SwinCVS trained end-to-end achieves 64.59% mAP and performs on par with SV2LSTG (64.68% mAP, p=0.470), while its pretrained variant achieves 67.45% mAP showing a significant improvement over the current SOTA.

**Conclusion:**

Our proposed solution offers a promising approach for CVS classification, outperforming existing methods and eliminating the need for semantic segmentation masks. Its design supports robust feature extraction and allows for future enhancements through additional tasks that force clinically relevant priors. The results highlight that attention-based architectures like SwinV2 are well suited for surgical image encoding, offering a practical approach for improving automated systems in laparoscopic surgery.

**Supplementary Information:**

The online version contains supplementary material available at 10.1007/s11548-025-03354-9.

## Introduction

Laparoscopic cholecystectomy (LC) is a surgical procedure to remove the gallbladder, performed in a minimally invasive manner and observed using a laparoscope. It is the fourth most common type of procedure carried out in England [1], with over 60,000 annual cases [2]. LC has become a widely adopted standard worldwide [3, 4], offering faster patient recovery and reduced treatment costs compared to open surgery [5]. However, operating through a laparoscopic camera poses its own challenges. It is reported that about 5$-$6.7% of operations performed in such a manner require patient readmission [4–6]. As a result, the high frequency of LC procedures combined with this readmission rate inevitably leads to many negatively affected patients [7, 8].

**Fig. 1:**
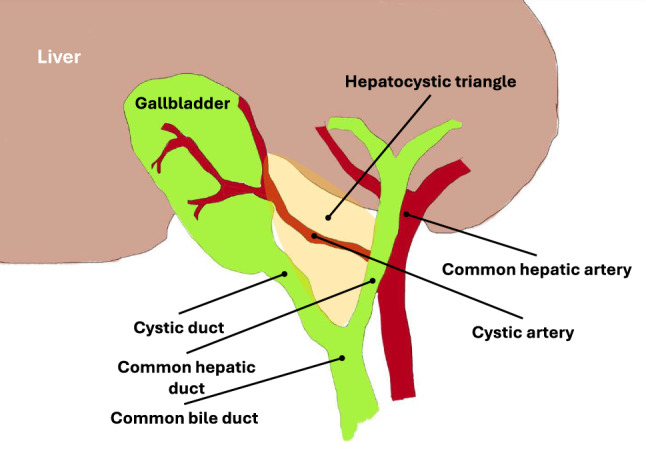
Anatomical diagram annotated with key structures associated with identification the critical view of safety. Hepatocystic triangle marked in yellow

The most serious type of complication occurs during the clipping phase of the operation, as the accidental transsection of incorrect bile and blood vessels can have a detrimental effect on patients and result in prolonged, life-changing consequences [4, 5, 9]. For this reason, the ‘critical view of safety’ (CVS) [10]-a method that facilitates intraoperative validation of the surgeon’s perception of anatomy-has been introduced and is now routinely used in standard practice [11]. This approach requires the clinician to confirm the presence of three independent anatomical structures before committing to any transsections. These are C1—clear dissection of the cystic duct and the cystic artery connecting with the gallbladder, C2—dissection of the hepatocystic triangle, C3— visual separation of the gallbladder from the cystic plate—a visualisation of which can be seen on Fig. 1. While the CVS has been shown to enhance patient safety by reducing the likelihood of surgical errors [11], reliably achieving this view during the operation can be challenging [12]. Consequently, an additional validation method utilising computer vision has been proposed to ensure the accurate identification of the CVS criteria.

The first CVS classification network, DeepCVS, was proposed in [13]. It utilised a segmentation network first to detect key anatomical structures and then combined their masks with the original image for classification using a convolutional neural network (CNN). Since then, several works have been published presenting similar models. Two notable examples are works published by Kawamura et. al. [14] and Wu et. al. [15]. Their respective investigations show promising results; however, both models were developed on private datasets limiting their reproducibility and direct comparison.

Recent progress in the field stems from the LatentGraph paper [16], where the authors innovated by combining segmentation with a graph network. This method constrained the model to focus on segmenting key anatomical structures relevant to CVS classification (cystic plate, hepatocystic triangle, cystic artery, cystic duct, gallbladder, tool) and spatially correlating them within a single image using a graph. By locally referencing each detected object, the approach enhanced image representation and improved overall classification performance. The subsequent work built on this by introducing temporal understanding, leading to the current state-of-the-art (SOTA) model, SV2LSTG [17].

Similarly to LatentGraph, SV2LSTG is a two-stage network consisting of a separately trained segmentation network followed by a graph neural network. The key distinction between the two models is that while LG-CVS uses a graph to map features within a single frame, SV2LSTG extends this by employing an additional graph encoder to correlate features across sequences of 10 frames, making it a spatiotemporal model. SV2LSTG is reported to achieve a 73.4% balanced accuracy score on the publicly available dataset ‘Endoscapes2023’. This dataset was introduced in [18] by the same research group and includes both CVS labels as well as semantic segmentation masks that were used to train both models.

While using a segmentation network to impose a prior on encoded features and applying pseudo-attention to spatially relate them has proven to be a significant advancement, the architecture still suffers from several limitations. Firstly, the sequential nature of the model, requiring multistage training, limits any further improvements of the architecture and risks error accumulation through optimising different parts of the network separately. Secondly, SV2LSTG only uses image information encoded into and extracted from segmented masks, preventing beneficial raw RGB images from influencing the classifier gradients during optimisation in the second stage. This limitation is clearly shown in [19], where classification scores are substantially lower between the model trained on the publicly available, smaller version of the dataset (50 videos - mAP = 64.3%) and the larger private version (201 videos - mAP = 69.7%). Finally, the absence of end-to-end training prevents the seamless integration of additional upstream or downstream tasks, such as incorporating depth maps alongside image data or adding additional tasks like segmentation, which could be used for constraining the model, thereby significantly enhancing the overall performance.

To address these limitations, we propose SwinCVS, a spatiotemporally aware network that outperforms existing models on the publicly available ‘Endoscapes2023’ dataset. SwinCVS supports end-to-end training, offering greater flexibility for future development. To summarise our contributions:

- Proposed a novel, real-time, spatiotemporal architecture for CVS classification that achieves state-of-the-art performance.
- Enabled end-to-end training that facilitates efficient feature extraction without relying on semantic segmentation masks.
- Evaluated three backbones: SwinV2, VMamba, and ResNet50, on an established CVS classification task.

## Methods

The primary objective of this work is to develop a spatiotemporal model with efficient image encoding, capable of end-to-end training for intraoperative CVS validation in LC. This is a multilabel classification task that focuses on predicting three distinct CVS criteria, C1-3, outlined in the introduction. Each frame from laparoscopic video can have none, some, or all (full CVS view) criteria visible at once. Experiments are divided into two parts: first, several backbone architectures are compared to assess their ability to encode image data. Next, the best-performing backbone is integrated with an LSTM to build the spatiotemporal model. This new architecture is evaluated under two settings: ‘Frozen’, where the backbone is pretrained on the target dataset, and ’End-to-End (E2E)’, where it is trained end-to-end with initialisation on ImageNet weights. The proposed model is directly compared to the current state-of-the-art SV2LSTG, with statistical tests used to assess performance.

All experiments are conducted on the Endoscapes2023 dataset [18], which comprises 201 videos of laparoscopic cholecystectomy, annotated for CVS detection, during the dissection phase. The dataset contains a total of 58,585 frames, of which 11,090 are annotated with binary CVS criteria labels, and 493 include semantic segmentation masks. A notable limitation of the dataset is the severe class imbalance, as only approximately 30% of the images display any visible CVS criteria. Full details regarding the splits between subsets can be found in Table 1, "Training Environment" section of the supplementary material.

**Table 1:** Table summarising the total number of frames per class in the training, validation and testing subsets

	Total	No CVS	C1	C2	C3
Training (120 videos)	6960	5023	1088	780	1245
Validation (41 videos)	2331	1757	381	291	389
Testing (40 videos)	1799	1031	423	302	501

C1, C2, and C3 correspond to respective CVS criteria. ‘No CVS’ refers to the lack of any visible CVS criteria

**Backbones**: The identification of individual CVS criteria requires not only the extraction of object-level features but also an understanding of their spatial relationships. For this reason, we compare three distinct backbone architectures: the SwinV2 transformer [20], the VMamba (visual space state model [21]), and the ResNet50 CNN [22], with the last one serving as our baseline.

SwinV2 was chosen as it leverages relative attention, which focuses on object relationships within localised regions of the image. While it lacks the global attention capabilities of models like Vision Transformer (ViT), its shifted window mechanism allows for dynamic adjustment of the attention area, enhancing its ability to capture local dependencies between objects. This focus on local relationships can be especially advantageous considering CVS classification given the requirement of detecting and encoding fine-grained structures such as blood vessels, which occupy only a small fraction of the image.

In contrast, VMamba employs a global attention mechanism with an optimised training strategy, achieving linear scalability without the quadratic complexity O($N^{2}$) seen in traditional transformer models. This reduction in computational cost enables efficient global attention, which in the case of CVS can better capture long-range dependencies across anatomical structures, potentially improving classification accuracy in challenging cases where local spatial information alone may be insufficient. However, this advantage comes with the trade-off that objects within the same patch may not fully benefit from the attention mechanism.

Finally, ResNet50 was chosen as a well-established CNN architecture because of its ability to create rich feature representations and effective mitigation of the vanishing gradient problem. Its convolutional feature encoding captures hierarchical representations of anatomical structures, enhancing the model’s ability to detect and classify objects relevant to CVS, and providing a reliable baseline for comparison with the attention-based models, SwinV2 and VMamba.

**Fig. 2:**
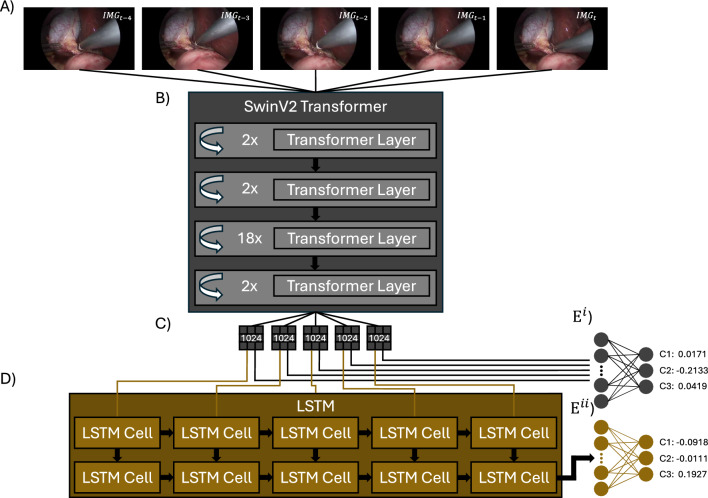
Visualisation of the SwinLSTM network. **A** Five-frame sequences are extracted from the dataset. **B** The SwinV2 Transformer encodes each image in the sequence individually. **C** Image encodings are retained in sequence, duplicated, and transmitted to both the encoder classifier and the LSTM. **D** The LSTM network establishes temporal correlations among the images; $E^{i}$ denotes the MLP classifier receiving individually encoded images from SwinV2, while $E^{ii}$ represents the MLP classifier utilising the output from the LSTM

**Spatiotemporal Architecture Overview**: The architecture is visualised in Fig. 2. The model begins by processing five images from the same sequence, encoding each one individually through the backbone. The encoded vectors are then split: one set is passed through a double-layer LSTM for temporal correlation followed by an MLP for CVS classification, while the other is fed into a mid-stream MLP classifier. We found that stacking two distinct architectures hindered gradient propagation, and the mid-stream MLP was able to improve the backbone’s optimisation.

The primary purpose of incorporating temporal comprehension is to enhance the model’s robustness against single-frame variations by leveraging information from multiple subsequent frames. This is accomplished through the LSTM’s capacity to maintain a hidden state $h_t$, which captures relevant temporal dynamics over time. The model predicts the label for the final frame in a five-frame sequence, reflecting the annotation practice used in the Endoscapes dataset, where images were labelled once every five frames. The proposed spatiotemporal model is evaluated through two independent pipelines. The ‘Frozen’ approach employs an Endoscapes pretrained backbone with fixed weights, optimising only the LSTM, while removing the midstream MLP. The ‘E2E’ approach initialises the backbone with ImageNet weights and fine-tunes both the image encoder and the LSTM on CVS data simultaneously, enabling end-to-end training. Following the backbone comparison detailed in the Results section, we selected SwinV2 for our spatiotemporal model, which will be referred to as SwinCVS in the subsequent discussion.

**Training Settings and Evaluation Metrics**: All backbone experiments were conducted with models initialised using pretrained weights from ImageNet and trained for 15 epochs. In the spatiotemporal model experiments, two versions of the model were evaluated, each trained for 10 epochs: the ’E2E’ version, which used ImageNet-initialised weights alongside a mid-stream MLP classifier, and the ’Frozen’ version, which employed the SwinV2 encoder initialised with the Endoscapes pretrained backbone. None of the backbones, nor SwinCVS utilised image augmentations. The SV2LSTG model was directly reproduced from the SurgLatentGraph GitHub repository [23] without any alterations.

The models were traied using the AdamW optimiser and evaluated on the Endoscapes2023 dataset, which included original train, validation, and test splits. Models were statistically compared using a one-tailed, paired t-test, assessing balanced accuracy (Bacc) and mean average precision (mAP) metrics. These metrics were chosen to provide complementary insights into model performance: mAP reflects the model’s ability to balance precision and recall across all CVS criteria, which is crucial for minimising missed detections that could lead to unsafe surgical conditions, while balanced accuracy evaluates the model’s consistency across all classes, ensuring that challenging or underrepresented criteria, such as the critical identification of the cystic artery and duct, are not disproportionately misclassified. Result scores were obtained on the testing hold-out subset and are presented as averages with sample standard deviations from five separate initialisation seeds. The stopping criterion was the best mAP score on the validation subset. All experiments were conducted on NVIDIA RTX A6000 graphics cards.

**Table 2:** Performance comparison for different backbones

Metric	SwinV2	VMamba	ResNet50
Bacc [%]	**67**.**45** ± **2**.61*	68.28 ± 1.64*	62.90 ± 0.51
mAP [%]	**64**.**52** ± **1**.93*$^{+}$	62.45 ± 1.71*	46.80 ± 1.63

Bold indicates the model selected for use as the backbone for our proposed architecture

Scores are average values between five independent initialisations on separate seeds, calculated on the testing hold-out set. Statistical significance $p<0.05$ over ResNet50 - $*$, over VMamba - $+$.

## Results

### Backbones

SwinV2 demonstrated the best performance for CVS classification, achieving a 64.52 mAP score, significantly outperforming both VMamba and ResNet50. Notably, although not reproduced in this paper, this score is comparable to that of the single-frame graph model LG-CVS (63.3% mAP) reported in [19] evaluated on the same dataset as ours. This highlights that SwinV2’s attention mechanisms effectively encode and reference spatial relationships of anatomical objects without requiring the expensive segmentation masks needed for training in both LG-CVS and SV2LSTG. For this reason, SwinV2 was chosen to use as a backbone in the spatiotemporal model experiments (Table 2).

**Table 3:** Performance comparison for spatiotemporal models

Model	CVS mAP [%]				CVS Bacc [%]			
	C1	C2	C3	Average	C1	C2	C3	Average
SwinCVS Frozen	65.02	61.38	75.95	67.45	71.22	68.89	70.62	70.25
SwinCVS E2E	64.23	62.50	67.03	64.59	70.14	66.29	64.19	66.88
SV2LSTG	68.71	59.72	65.82	64.68	78.04	75.25	70.98	74.76

Scores are average values between five independent initialisations on separate seeds, obtained on the testing hold-out set

### Spatiotemporal models

Our SwinCVS Frozen model demonstrated the strongest performance, achieving an mAP of 67.45% and a Bacc of 70.25% (Table 3). Notably, it showed a statistically significant improvement in mAP over SV2LSTG (p < 0.05), though SV2LSTG outperformed in Bacc, achieving 74.76%, with a significant difference from both SwinCVS Frozen and SwinCVS E2E (p < 0.05). The SwinCVS E2E model’s mAP closely matched that of SV2LSTG, with no significant statistical difference observed between them for this metric. Given that the mAP score is the distinguishing factor when comparing the models in [19], we can conclude that although SV2LSTG achieved a higher Bacc, our proposed architecture delivers equal or superior performance, while retaining all the previously mentioned advantages—notably, only relying on weakly labelled images.

The full results table with runs on different initialisations for both backbones and spatiotemporal experiments can be found in the supplementary material, “Results Discussion” section.

## Discussion

Our experiments analysed several backbones and compared our proposed SwinCVS model against the current SOTA. We selected the mAP metric for direct comparison, as it offers a more comprehensive evaluation than Bacc by capturing the precision-recall trade-off across multiple thresholds. This makes mAP particularly well-suited for high-level performance comparisons between models, while Bacc serves to assess a model’s ability to differentiate between No CVS and CVS on a case-by-case basis.

In terms of backbones, SwinV2 demonstrated superior performance, significantly improving over both VMamba (+2.07% mAP) and ResNet50 (+17.72% mAP). A likely reason for VMamba’s suboptimal performance lies in its global attention mechanism. While this can be beneficial in certain contexts, it may struggle with CVS criteria, especially when small, proximate structures are involved. VMamba’s static patching approach limits its ability to differentiate between objects within the same patch, potentially overlooking finer local details like the cystic artery and duct. In contrast, SwinV2’s shifted window mechanism dynamically adjusts its attention, likely allowing for better encoding of local spatial relationships and improving the model’s precision in distinguishing critical CVS structures.

By reviewing the results of our SwinCVS and SV2LSTG networks, focusing on mAP scores, we can conclude that our proposed architecture matches (64.59%$\approx$64.68% mAP, p=0.47), and in the case of Frozen variant, exceeds the current SOTA solution (+2.77% mAP, p<0.05). It is important to note that E2E achieved results comparable to SV2LSTG, despite being trained solely on weakly labelled images, while SV2LSTG relies on costly semantic segmentation masks. This demonstrates the added benefit of the attention mechanisms employed by SwinV2, making it more efficient at extracting and encoding valuable information from the images.

**Fig. 3:**
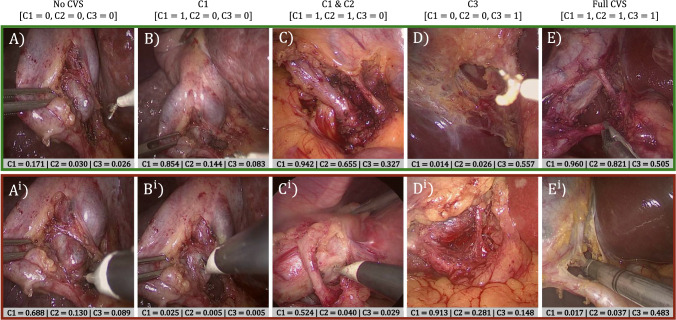
Confidence evaluation of the SwinV2 E2E model on images with different CVS labels. Top row shows correctly classified images and the bottom one, indicated with $\hbox {X}^{i}$, incorrectly. Each case is associated with the model’s prediction confidence

Considering differences between backbone and SwinCVS results we can see that the improvement oscillates around +2.93% mAP points. This is a higher improvement to what authors achieved in [19], when moving from single-frame LG-CVS to temporal SV2LSTG (+1.1%), despite SwinCVS using a simpler LSTM architecture instead of a complex graph network.

One area where SwinCVS underperforms relative to the SV2LSTG is in the classification of the C1 criterion - representing a clear dissection of the cystic artery and duct. This limitation likely arises from the absence of explicit spatial supervision such as segmentation masks, which restricts its ability to capture the subtle anatomical details required for C1 classification. Furthermore, the inherent visual ambiguity of the cystic duct and artery, which are small structures often partially occluded and closely resemble surrounding tissues, poses significant challenges for weakly supervised models like SwinCVS. In contrast, the SV2LSTG additionally leverages specific encoding of spatial relationships from segmentation maps, providing an advantage in detection of such anatomies.

Figure 3 analyses the classification performance of our proposed SwinCVS architecture (E2E variant) on individual image examples, using a standard classification threshold of 0.5. The model demonstrates good robustness even in the presence of blurred images, as illustrated in 3.B. It successfully identifies instances with drastic variations in lighting, exemplified by image 3.E. In all misclassification cases, there are reasonable explanations for the model’s confusion; for instance, in example 3.$\hbox {A}^{i}$, the cystic artery is in the process of being dissected, and in 3.$\hbox {E}^{i}$, both the hepatocystic triangle and vessels are obscured by the grasper. However, the model often struggles with inherently ambiguous cases. In the Endoscapes dataset, annotators frequently disagree, resulting in a true label of e.g. 0.33 or 0.66 (rounded to 1 during training). Both images 3.$\hbox {A}^{i}$ and 3.$\hbox {D}^{i}$ are examples of such occurance. This raises the possibility that the model’s confusion may stem not only from the model’s performance but also from the varying interpretations of the three annotating surgeons. Given these examples, it is clear that SwinCVS provides reasonable estimations regarding the presence of CVS criteria based on the anatomical context. Consequently, we recognise that further refinement of the model will require careful consideration of trade-offs, particularly in balancing accuracy with clinical relevance.

The inference times of SwinCVS and the SwinV2 backbone were also analysed. Both models demonstrated real-time performance on an Nvidia A6000 GPU, achieving approximately 9 Hz (107.8±8 ms per frame) and 25 Hz (40.5±9.4 ms per frame) - indicating they are viable for use in time-critical applications such as CVS detection (Table 4). Inference time does not change between ’Frozen’ and ’E2E’ variants of the SwinCVS model.

**Table 4:** Inference time for SwinV2 backbone and spatio-temporal SwinCVS models

Model version	Mean (ms)	Std (ms)
SwinV2 Backbone	40.45 (24.72 Hz)	9.43
SwinCVS	107.86 (9.27 Hz)	8.07

Our study also found a notable performance improvement observed in the Frozen model compared to its E2E-trained counterpart. In theory, fully end-to-end trained models have an advantage by being able to holistically optimise all of their parameters in all stages of the network for a given inference task. In the case of SwinCVS, this allows the E2E model to also optimise for the detection of temporal cues in the encoder. In practice, this substantially increases the complexity of the training routine, requiring careful consideration of different learning rates at different stages of the network, or changes to the model architecture like the adoption of multiple classifiers (e.g. Fig 2). Therefore, where E2E models might struggle with overfitting, the Frozen variant allows the model to focus on one particular task at a single point in time, artificially implementing a regularisation effect.

Currently, the architecture is trained exclusively on weakly labelled images, which limits its performance and the capacity to visually explain the results. Additionally, the model’s optimisation is centred on the mAP metric, which deprioritises Balanced Accuracy - a limitation that could be addressed through adjusted training parameters. To improve its robustness in challenging classification scenarios, further fine-tuning is required. This could potentially be achieved by replacing the MLP classifier following the encoder with an object detection or segmentation network, directing the model’s focus towards predefined, clinically relevant anatomical features.

## Conclusion

In this paper, we introduced SwinCVS, a spatiotemporal, real time, architecture for classifying CVS in laparoscopic cholecystectomy images. Our model leverages the SwinV2 image encoder, which has been empirically validated as the most effective backbone for this task. A comparative experiment with SwinV2, VMamba, and ResNet50 showed that SwinV2’s shifted window attention is particularly well-suited for capturing the spatial relationships critical to CVS classification.

SwinCVS matches the performance of the current state-of-the-art (SV2LSTG) when trained end-to-end and surpasses it when pretrained on the target dataset. Crucially, SwinCVS achieves these results without requiring semantic segmentation masks, highlighting its more efficient processing of image data. Furthermore, the end-to-end nature of our model allows for straightforward modifications, such as the incorporation of auxiliary tasks that can enforce clinically relevant priors, e.g. anatomical structure awareness through image reconstruction. Finally, our evaluation also showed that the misclassifications made by SwinCVS are reasonable and often arise from the inherent ambiguity and difficulty of the CVS labels within the dataset.

In summary, SwinCVS provides an effective approach to CVS classification, improving existing methods and achieving SOTA performance. Our experiments show that attention-based architectures like SwinV2 are better suited for encoding surgical images compared to graph-based models, and the flexibility of SwinCVS makes it a solid foundation for future research.

## Supplementary Information

Below is the link to the electronic supplementary material.

Supplementary file 1 (pdf 553 KB)

## Data Availability

This publication did not require ethical approval. Dataset is publicly available [18], while corresponding code is available at https://github.com/franeknowak/SwinCVS, for research purposes.
