# Supplementary material for: SwinCVS: a unified approach to classifying critical view of safety structures in laparoscopic cholecystectomy
Source: Int J Comput Assist Radiol Surg. 2025 Apr 11;20(6):1145–52. doi: 10.1007/s11548-025-03354-9 (PMC12167293; doi:10.1007/s11548-025-03354-9)
Supplement: Supplementary file 1 — (pdf 553 KB) [file 11548_2025_3354_MOESM1_ESM.pdf]

Supplementary materials for submission: ”SwinCVS: A Unified Approach to Classifying Critical View of Safety Structures in Laparoscopic Cholecystectomy”

---

1 Training Environment

Label Subset	Training	Validation	Testing
All images	6960	2331	1799
No CVS	5023	1757	1031
C1 Total	1088	381	423
C2 Total	780	291	302
C3 Total	1245	389	501
C1 Excl.	481	95	165
C2 Excl.	73	19	21
C3 Excl.	576	143	260
C1 & C2	138	71	81
C1 & C3	100	45	41
C2 & C3	200	31	64
Full CVS [C1 & C2 & C3]	369	170	136

Table 1: Number of annotated CVS frames per class in the Endoscapes Dataset.

2 Results details

MAP / BACC T.Test	Frozen	E2E	SV2LSTG
**Frozen**	x / x	0.046 / 0.276	0.000 / 0.009
**E2E**	0.046 / 0.276	x / x	0.000 / 0.001
**SV2LSTG**	0.000 / 0.009	0.000 / 0.001	x / x

Table 2: Statistical comparison between different backbone models.

MAP / BACC T.Test	Frozen	E2E	SV2LSTG
**Frozen**	x / x	0.023 / 0.159	0.048 / 0.002
**E2E**	0.023 / 0.159	x / x	0.470 / 0.043
**SV2LSTG**	0.048 / 0.002	0.470 / 0.043	x / x

Table 3: Statistical comparison between different spatio-temporal models.

		CVS mAP [%]				CVS Bacc [%]			
**Model**	**Seed**	**C1**	**C2**	**C3**	**Average**	**C1**	**C2**	**C3**	**Average**
**SwinV2**	0	67.45	57.95	68.50	64.63	69.00	66.48	69.17	68.22
	1	64.11	50.37	69.47	61.32	76.98	69.00	65.22	70.40
	2	66.49	62.22	70.03	66.25	73.14	63.50	65.45	67.36
	3	64.07	58.64	71.11	64.61	71.22	60.12	58.44	63.26
	4	65.51	63.48	68.40	65.80	70.47	68.86	64.76	68.03
	**Average**	**65.53**	**58.53**	**69.50**	**64.52**	**72.16**	**65.59**	**64.61**	**67.45**
	**Stdev**	**1.48**	**5.12**	**1.13**	**1.93**	**3.08**	**3.79**	**3.87**	**2.61**
**VMamba**	0	62.95	64.35	67.25	64.85	70.27	74.68	67.99	70.98
	1	57.55	54.28	69.65	60.49	62.64	70.01	67.75	66.80
	2	59.05	60.34	66.59	61.99	72.72	70.25	61.69	68.22
	3	61.01	60.27	69.06	63.45	66.34	69.26	65.78	67.13
	4	63.06	54.80	66.60	61.49	68.52	69.44	66.92	68.29
	**Average**	**60.72**	**58.81**	**67.83**	**62.45**	**68.10**	**70.73**	**66.03**	**68.28**
	**Stdev**	**2.42**	**4.24**	**1.43**	**1.71**	**3.85**	**2.25**	**2.57**	**1.64**
**ResNet50**	0	50.53	46.14	49.40	48.69	63.53	63.78	63.17	63.49
	1	47.52	46.32	50.52	48.12	63.50	62.83	61.93	62.75
	2	42.82	43.77	47.43	44.67	61.53	62.49	62.46	62.16
	3	45.39	43.03	49.30	45.91	63.33	62.92	62.31	62.85
	4	44.20	45.15	50.53	46.63	63.43	62.82	63.41	63.22
	**Average**	**46.09**	**44.88**	**49.44**	**46.80**	**63.06**	**62.97**	**62.66**	**62.90**
	**Stdev**	**3.02**	**1.45**	**1.27**	**1.63**	**0.86**	**0.48**	**0.62**	**0.51**

Fig. 1: Full numerical results for backbones.

		CVS mAP [%]				CVS Bacc [%]			
**Model**	**Seed**	**C1**	**C2**	**C3**	**Average**	**C1**	**C2**	**C3**	**Average**
**Frozen**	0	64.41	62.30	74.13	66.94	72.34	69.65	73.78	71.92
	1	64.68	59.09	78.51	67.43	70.24	67.23	72.63	70.03
	2	66.24	63.62	73.84	67.90	72.55	68.36	64.64	68.52
	3	65.33	61.21	76.91	67.82	70.06	69.22	68.74	69.34
	4	64.45	60.66	76.37	67.16	70.92	70.00	73.33	71.42
	**Average**	**65.02**	**61.38**	**75.95**	**67.45**	**71.22**	**68.89**	**70.62**	**70.25**
	**Stdev**	**0.77**	**1.71**	**1.96**	**0.41**	**1.16**	**1.11**	**3.89**	**1.42**
**E2E**	0	64.45	66.83	62.85	64.71	76.83	74.47	66.66	72.65
	1	67.65	66.95	68.53	67.71	71.91	60.43	54.04	62.13
	2	64.58	59.98	65.53	63.36	62.57	51.32	54.01	55.97
	3	66.00	60.59	69.88	65.49	69.72	72.57	75.89	72.73
	4	58.49	58.13	68.36	61.66	69.68	72.68	70.36	70.91
	**Average**	**64.23**	**62.50**	**67.03**	**64.59**	**70.14**	**66.29**	**64.19**	**66.88**
	**Stdev**	**3.46**	**4.11**	**2.82**	**2.27**	**5.14**	**10.07**	**9.85**	**7.51**
**SV2LSTG**	0	71.73	57.51	65.11	64.78	77.08	74.47	68.68	73.41
	1	71.46	63.28	68.83	67.86	79.54	76.75	72.49	76.26
	2	63.49	63.64	57.03	61.05	77.05	76.00	68.61	73.89
	3	66.71	53.26	70.70	63.56	77.00	72.26	72.61	73.96
	4	70.16	60.89	67.44	66.16	79.54	76.75	72.49	76.26
	**Average**	**68.71**	**59.72**	**65.82**	**64.68**	**78.04**	**75.25**	**70.98**	**74.76**
	**Stdev**	**3.54**	**4.36**	**5.32**	**2.59**	**1.37**	**1.91**	**2.13**	**1.39**

Fig. 2: Full numerical results for spatiotemporal models.
